# Nailfold Videocapillaroscopy in Patients with Central Serous Chorioretinopathy and Its Relationship to Morphological and Functional Findings

**DOI:** 10.3390/jcm9123891

**Published:** 2020-11-30

**Authors:** Małgorzata Latalska, Joanna Bartosińska, Ewa Kosior-Jarecka, Dorota Krasowska, Jerzy Mackiewicz

**Affiliations:** 1 Department of Vitreoretinal Surgery, Medical University of Lublin, 20-079 Lublin, Poland; okulistyka.retina@umlub.pl; 2 Department of Cosmetology and Aesthetic Medicine, Medical University of Lublin, 20-093 Lublin, Poland; jbartosinski@gmail.com; 3 Department of Diagnostics and Microsurgery of Glaucoma, Medical University of Lublin, 20-079 Lublin, Poland; ewakosiorjarecka@umlub.pl; 4 Department of Dermatology, Venereology and Pediatric Dermatology, Medical University of Lublin, 20-093 Lublin, Poland; dorotakrasowska@umlub.pl

**Keywords:** videocapilaroscopy, central serous chorioretinopathy, microcirculation

## Abstract

The aim of the study was to evaluate the results of nailfold videocapillaroscopy (NVC) in patients with central serous chorioretinopathy (CSC) and their correlation with different functional, morphological features and general risk factors. Material and methods: The examined group included 59 CSC patients (14 F, 45 M, mean age 47.2 ± 9.4) and 53 healthy controls (13 F, 40 M, mean age 46 ± 11.5). The NVC was qualified as a normal or abnormal pattern. In the CSC group, the ophthalmoscopy, angio-Optical Coherence Tomography (OCT-A), OCT and microperimetry were performed. The medical history regarding CSC, chronic general disorders and known risk factors was recorded. Results: In the CSC group an abnormal NVC pattern was more common (33.88% CSC vs. 7.54%, *p* < 0.001). Dilated apical part of capillaries, microaneurysmal dilatation, megacapillaries, fresh microhaemorrhages, bizarre and broken capillaries were observed only in CSC patients. Dilation of capillaries (47.56% vs. 13.21%, *p* = 0.004), ramified capillaries and neoangiogenesis (38.98% vs. 5.66%, *p* < 0.001), glomerular loops (32.20% vs. 1.88% *p* < 0.001) were more frequent in the CSC group. Conclusions: The presence of various NVC abnormalities confirms microvascular involvement in CSC pathogenesis. The results correlate with visual acuity, microperimetry, OCT data and stress. The NVC technique may play a useful diagnostic and prognostic role in CSC.

## 1. Introduction

Central serous chorioretinopathy (CSC) is a chorioretinal disease, one of pachychoroid disorders, that primarily affects men aged 20–60 years. In its course visual loss is due to idiopathic serous detachment of the retina, associated with one or more regions of leakage from the choroid. It is induced by a defect in the retinal pigment epithelium (RPE)—the outer blood–retina barrier. The cause of this disease still remains unclear [1,2]. However, color Doppler ultrasonography revealed hemodynamic changes in the ophthalmic artery, reflecting choroidal hyperperfusion and central choroidal thickness measured by Optical Coherence Tomography (OCT) [1,2]. Toslac et al. found that hemodynamic changes in the ophthalmic artery (OA) reflected choroidal hyperperfusion. Hemodynamic parameters (as resistive index (RI), pulsatility index (PI) and peak systolic velocity (PSV)) of the OA and (PSV, end-diastolic velocity (EDV), as well as mean velocity (Vmean)) the CRA were lower in the CSC patients than in the control group. On the other hand, RI and PI of the CRA were greater in the CSC group compared to the control group. Moreover, the central choroidal thickness (CCT) was significantly greater in the affected eyes of the CSC group compared to the unaffected eyes in the CSC and control groups [1]. Kilic et al. also assessed the posterior ciliary arteries (PCA). They revealed a negative correlation between submacular choroidal thickness (SCT) and peak systolic velocity PSV of PCA and between SCT and end-diastolic velocity EDV of PCA in CSC patients. They found an ocular circulatory dysfunction in patients with CSC [2].

The additional examination, evaluating microcirculation disturbances, may be capillaroscopy. Nailfold videocapillaroscopy (NVC) is one of the best diagnostic imaging techniques for studying the microcirculation in vivo. It is a noninvasive, repeatable, simple and inexpensive method, which permits assessment of the microcirculation directly [3]. It is widely used in the diagnostic of microvascular pathologies in dermatologic and rheumatologic conditions, as well as in diabetes mellitus (DM) and arterial hypertension. The NVC may aid in early diagnosis of the connective tissue disorders and in the differential diagnosis of primary Raynaud phenomenon (RP) from secondary RP due to systemic scleroderma (SSc) and mixed connective tissue disease (MCTD). In comparison to standard nailfold capillaroscopy, it has such advantages as: real-time control of the image obtained, precision of image storage and reproduction and advanced image analysis and measuring features. A further advantage is the presence of a contact probe with polarized light microscopy allowing easier observation of the skin surface [4]. NVC is becoming more and more popular also in ophthalmology, especially in the assessment of microcirculation in glaucoma [5,6]. However, there is only one study evaluating the results of NVC in CSC that concerns the relationship with choroidal thickness [7].

The aim of the current study was to evaluate the results of the NVC in patients with CSC in comparison to gender and age-matched healthy controls (HCs). In addition, the correlation between the results of NVC examination and clinical status and observed risk factors of CSC group were also analyzed.

## 2. Experimental Section

### 2.1. Materials and Methods

#### 2.1.1. The Study Groups

The study group consisted of 59 patients with CSC, treated in Department of Vitreoretinal Surgery, Medical University in Lublin, Poland in years 2018–2020 and 53 healthy, gender and age-matched healthy control (HCs). Written consent was obtained from all the participants before the enrollment in the study. The study adhered to the tenets of the Declaration of Helsinki and the design was approved by the local ethical committee in Medical University of Lublin (KE-0254/291/2018).

The study group consisted of 14 female and 45 male (mean age 47.2 ± 9.4) CSC patients.

The inclusion criteria to CSC group were:

1/The active CSC was defined as a serous detachment of the neuroretina (diagnosis of CSC was confirmed after clinical and optical coherence tomography examination (OCT)) [8]. Additionally, OCT angiography (OCT-A) was performed to exclude subretinal, macular neovascularization (MNV).

The exclusion criteria from CSC group were as follows: 1/autoimmune diseases such as Ssc and systemic lupus erythematosus (SLE) due to their significant impact on NVC scores, 2/general vascular (e.g., peripheral artery disease, abdominal aortic aneurysm, carotid artery disease, pulmonary embolism, chronic venous insufficiency, anemia) and neoplastic disorders, 3/history of anticancer treatment, 4/debilitating conditions such as chronic alcoholism or drug addiction, 5/concurrent ocular and retinal disease affecting visual acuity, including diabetic retinopathy, age-related macular degeneration, vitreomacular disorders, presence of RPE atrophy and presence of cystic degeneration on structural OCT.

The HCs group was examined, as described later, to exclude any ocular diseases. That group included 13 female and 40 male (mean age 46 ± 11.5) healthy volunteers. The study and control groups did not significantly differ in age and gender (*p* = 0.51 and *p* = 0.39, respectively). Demographic data of CSC and HCs are presented in Table 1.

#### 2.1.2. Ophthalmologic Examination

All participants (CSC patients and HCs) underwent: best corrected distal (BCDVA) and near visual acuity (BCNVA) examination (using Snellen charts), fundus ophthalmoscopy, spectral-domain SD-OCT imaging (using SOCT Copernicus HR, Optopol). The OCT scans were analyzed when the signal quality was between 8 and 10. The OCT images of CSC patients were reviewed for qualitative features and analyzed for quantitative measures of central retinal thickness, subfoveal subretinal fluid height (SRF), pigment epithelium detachments (PEDs), ellipsoid zone disruption and presence of intraretinal hyperreflective foci (HF). The central retinal thickness (CRT) was measured in a horizontal section image of OCT in the fovea as a distance between internal limiting membrane and RPE-Bruch’s membrane complex, SRF height was measured in the fovea as a distance between the top of the SRF and the RPE-Bruch’s membrane complex. The presence of morphological changes of the retina, in form of alteration in RPE layer, PED, HF and the state of the ellipsoid zone (EZ) were assessed. All CSC patients had a microperimetry examination (MAIA, Centervue)—evaluating average retinal sensitivity, average threshold (AT) and macular integrity (MI). The known risk factors for CSC like steroid, xylometazoline and phosphodiesterase inhibitor intake, diabetes mellitus, autoimmunological diseases were assessed in reports.

#### 2.1.3. Nailfold Videocapillaroscopy in the CSC Patients and HCs

NVC was conducted in the Department of Dermatology, Venereology and Pediatric Dermatology, Medical University, Lublin, Poland. In all CSC patients and HCs the NVC was performed by an experienced dermatologist, blinded to clinical data (JB) with the use of a videocapillaroscopy VideoCap 3.0 (DSMedica) at 200× magnification.

Before the NVC examination, every patient was asked about the symptoms of hands freezing.

The examination was carried out according to a standard protocol. All participants were asked to fast, avoid smoking, avoid drinking alcohol and caffeinated drinks and avoid taking any drugs that could affect the circulatory system 24 h prior to the examination. They were also instructed not to remove fingernail cuticles for one month before the examination. The examination was performed in the room temperature of 21–23 °C and then patients waited for 15–20 min. A drop of immersion oil was placed on each examined finger cuticle to facilitate visibility. During NVC the patient was in sitting position with his/her hands at the heart level.

The nailfold capillaries in the second to fifth fingers of both hands were examined. On NVC the following parameters were evaluated: capillary distribution (i.e., capillary architecture), capillary length, capillary morphology (i.e., meandering capillaries, coiled capillaries, tortuous capillaries, bushy-ramified capillaries and angiogenesis), capillary diameter (i.e., dilated capillaries (>20 µm), dilated apical part of capillaries, capillary enlargement (30–50 µm), megacapillaries (>50 µm), capillary density (normal 9–14 capillaries per mm), the presence of microhemorrhages, microaneurysms and visibility of subpapillary venular plexus.

Normal capillary architecture referred to the presence of homogeneous distribution of capillaries arranged in parallel in the distal row of the nailfold. Disorganization of the capillary architecture was defined as irregular capillary distribution, orientation and morphology.

After the detailed description of the nailfold capillaries, the obtained results were interpreted as a normal or abnormal NVC pattern. Normal NVC pattern was described as: normal capillary architecture, normal capillary density (9–14 capillaries per millimeter) and the presence of ≤2 tortuous/coiled capillaries per millimeter and/or ≤2 meandering capillaries per millimeter and/or ≤2 bizarre loops per millimeter and/ or ≤1 ramified capillary per millimeter.

Abnormal NVC pattern was defined as follows: disorganization of the capillary architecture, and/or >2 tortuous/coiled capillaries per millimeter and/or >2 meandering capillaries per millimeter and/or >2 bizarre loops per millimeter and/or >1 ramified capillary per millimeter and/or >2 dilated capillary (diameter 20–50 µm) and/or decreased capillary density and/or the presence of at least one megacapillary, broken capillary, ramified capillary or fresh microhemorrhage.

All results were compared to the “scleroderma pattern” in order to exclude patients suspected with undiagnosed SSc [9,10].

#### 2.1.4. Statistical Analysis

Statistical analysis was conducted with Statistica 12 software, and *p* ≤ 0.05 was considered statistically significant. The results were reported mainly as percentage values, in this case analysis was performed as the difference between structures of the indicators. Normal distribution was verified with the Shapiro–Wilk test. In the case of non-normally distributed data, the ANOVA Kruskal–Wallis test with Tukey was used as a post-hoc test. Chi-square test with Yates modification when needed was used to check the association between risk factors of CSC and NVC results. In the case of statistical significance obtained in Chi-square test, logistic regression analysis was performed to obtain p and assess odds ratios (ORs). Correlations were assessed with the use of Spearman test.

## 3. Results

### 3.1. Comparison of NVC Pattern between CSC and HCs

Abnormal NVC pattern was found in 20 (33.89%) of 59 CSC patients. In HCs, abnormal NVC results were observed only as abnormal distribution of capillaries in 4 cases (7.54%) (abnormal NVC pattern CSC vs. HCs: *p* = 0.007). Abnormal distribution of capillaries was observed in 12 (20.34%) of the CSC patients. Enlargement of capillaries >30 µm was found in 22 (37.29%) CSC patients and no HCs (*p* = 0.007). In the CSC group dilated apical part of capillaries (28.81%), aneurysmal dilatation (10.17%), bizarre loops (5.08%), broken capillaries (5.08%), megacapillaries (1.6%) and fresh hemorrhages (3.39%) were found and were not observed in the HCs. Hands freezing was observed only in CSC patients (11.86%). Capillaroscopic findings including abnormal NVC pattern (33.89% CSC vs. 7.54% HCs, *p* = 0.000), dilation of capillaries (47.56% vs. 13.21%, *p* = 0.004), ramified capillaries and neoangiogenesis (38.98% vs. 5.66%, *p* = 0.000) and glomerular loops (32.20% vs. 1.88%, *p* = 0.000) were significantly more frequent in CSC group.

**Figure 1:**
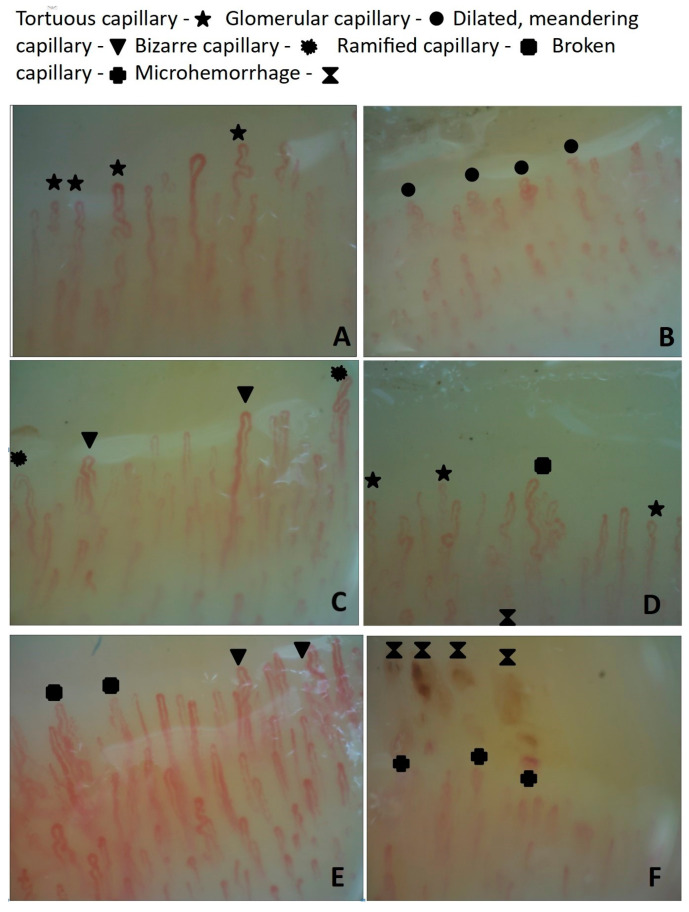
Capillary changes in patients with central serious chorioretinopathy (CSC): (**A**). Tortuous capillaries; (**B**). Glomerular capillaries; (**C**). Dilated, meandering and bizarre capillaries; (**D**). Tortuous, ramified capillaries, neoangiogenesis; (**E**). Dilated, meandering and ramified capillaries; (**F**). Abnormal distribution, broken capillaries, microhemorrhages.

Capillary changes in patients with central serous chorioretinopathy are presented in Figure 1.

### 3.2. Relationship between NVC Results and Ophthalmic Features in CSC Group

There was no correlation between NVC findings and most commonly observed OCT features like RPE alteration, PED, SRF height and CRT. The OCT examination revealed RPE alteration in 45 cases (76.27%), PED in 11 (18.64%), HF in 39 (66.1%) and EZ disruption in 11 (18.64%) patients.

The comparison of NVC results in CSC and HCs is presented in Table 2. Correlations between NVC findings, morphological and functional results are presented in Table 3 and Table 4.

In logistic regression analysis, enlargement of capillaries and glomerular loops were shown to be significant for HF on OCT (OR, 6.8; *p* = 0.025 and OR 0.02; *p* = 0.013, respectively). Additionally, hand freezing was significant for EZ disruption (OR, 9.6; *p* = 0.025).

### 3.3. Correlations between NVC Pattern and General Risk Factors and OCT Features

The mean duration of symptoms reported by patients was 13.48 ± 22.8 months (range from 0.5 to 120 months). Duration of symptoms reported by patients was significantly correlated with abnormal NVC pattern (r 0.5) and dilated apical part of capillaries (r 0.3), and OCT features like EZ disruption (r 0.4), SRF height (r −0.4), BCDVA (r −0.4) and CRT (r −0.4).

In our study, stress and smoking cigarettes occurred to be significantly related to some NVC results. Occupational stress was revealed in 13 patients (22.03%) and was significantly more common in cases with dilated apical part of capillaries (*p* = 0.02). Patients reporting occupational stress in their lives had significantly worse AT (*p* = 0.03). Stress was also strictly correlated with dilated apical part of capillaries (r 0.3, OR 4.2, *p* = 0.034) and BCDVA (r −0.3, OR 0.02, *p* = 0.04). BCDVA and MI were significantly better in non-smoker (*p* = 0.03 and *p* = 0.03, respectively). The CSC group included 9 smokers (15.25%). A significant positive correlation was found between smoking and tortuous loops, as well as megacapillaries (r 0.3, OR 0.11, *p* = 0.12; r 0.3, OR 2.9, *p* = 1.0, respectively). Megacapillaries were significantly more frequent in smoking patients compared to nonsmokers (*p* = 0.02) (Table 5). General diseases and risk factors are presented in Table 1. Correlation between general risk factors and NVC results is presented in Table 5.

## 4. Discussion

NVC is used to evaluate disturbances in the skin microvasculature of patients with autoimmune connective tissue disorders, especially in SSc. Local vascular abnormalities in the nailfold capillaries seem to appear earlier in the course of these diseases than at other sites of the skin. The most characteristic outcomes occur in SSc, dermatomyositis, and MCTD as well as in syndromes that overlap with SSc [9]. Sclerodermia pattern” includes the presence of certain abnormalities or their combination, i.e., the presence of giant capillaries (“giants”) or the combination of abnormal shapes (i.e., ramifications, branching, bushy or coiled capillaries) with an extremely lowered number of capillaries. In SSc irregular capillary distribution and orientation, enlargement of capillaries as well as microhemorrhages are commonly observed [9].

In the present study, dilation of capillaries (diameter > 20 µm) was significantly more common in CSC group than in HCs while enlargement of capillaries (diameter 30–50 µm) and dilated apical part of capillaries were observed only in CSC group. Furthermore, capillary microhemorrhages, and some of the structural abnormalities in NVC (i.e., aneurysmal dilatations, broken capillaries, bizarre capillaries) were detected only in the CSC group. Ramified capillaries, glomerular and meandering capillaries were significantly more common in patients with CSC, but they were also present in the HCs. All these symptoms represent characteristic elements of microvascular involvement and dysregulation of blood vessels due to impaired autoregulation of blood flow. It seems plausible that abnormalities in nailfold capillaries of CSC patients result from vasoconstriction in the microcirculation and suggest a tendency to vasospasm in this disease. Interestingly, 11.83% CSC patients reported hands freezing which is considered to be one of the risk factors of the vascular disorders. Yavaş et al. noticed more frequent occurrence of obstructive sleep apnea (OSA) in male CSC patients which might suggest the involvement of vasoconstriction in the pathogenesis of CSC [11]. However, the role of NVC in CSC patients still awaits elucidation. The only available study assessing NVC in CSC revealed capillary ectasia, aneurysm, microhemorrhage, avascular area, tortuosity, neo-formation, bizarre capillary, bushy capillary, meander capillary and extravasation, but without significant relations with choroid thickness and CSC type (acute or chronic) [7].

In the study we found some interesting correlations between NVC results, as well as morphological and functional outcomes. There was significant correlation between hand freezing and dilated apical part of capillaries with EZ disruption, evidence of photoreceptors impartment, and which may additionally support possible relation with microvascular dysregulation in CSC pathogenesis. Additionally, hand freezing, the effect of autoregulation disturbances, may itself indicate the participation of unstable microcirculation in the pathogenesis of CSC.

NVC changes as an enlargement of capillaries, glomerular loops, meandering loops, ramified capillaries and neoangiogenesis correlated significantly with HF. Hyperreflective foci was previously described in CSC and was proposed as an independently significant predictor for macular SRF complete resolution at 3 months [12]. It was suggested that HF constitute the extravasation of lipoproteins or activated microglia with a phagocytized photoreceptor or intraretinally migrated RPE. Moreover, the baseline number of HF may be able to predict the course of anatomical and functional recovery, as well as the recurrence of CSC by Lee et al. [13]. Accordingly, the presence of these HF may reflect greater damage of the RPE and worse prognosis for visual acuity. Thus, glomerular loops, neoangiogenesis, meandering loops and enlarged capillaries, significantly correlated with HF, may be used to predict RPE damage and final visual acuity, especially when negative significant correlation between HF and BCDVA, as well as neoangiogenesis and BCDVA were found in our study. Further studies are needed to shed more light on this hypothesis.

A significant negative correlation was found between the dilated apical part of capillaries and neoangiogenesis with BCDVA. This may suggest that presence of these NVC features is correlated with worse visual outcomes. What is more, the correlations between some NVC results and functional features (BCDVA, BCNVA, AT and MI) may indicate the possible role of NVC examination in predicting the visual recovery. All these relationships may allow the assumption that the presence of glomerular and tortuous loops seems to be beneficial for patients with CSC, but dilated apical part of capillaries and neoangiogenesis worsen the prognosis for visual acuity. Glomerular loops, dilated apical part and neoangiogenesis were significantly more common in CSC group, but not the tortuous loops. Therefore, we suggest that glomerular loops, dilated apical part and neoangiogenesis could become prognostic factors of visual acuity in patients with CSC. Furthermore, dilated apical parts and hand freezing were significantly correlated with EZ disruption, and neoangiogenesis with HF presence. In our opinion, such abnormalities could play a part in the characterization of CSC microangiopathy. There is a possibility that NVC could be used to detect patients predisposed to worse prognosis for visual outcome. Thus, NVC findings can supply useful information as to pathophysiology, differential diagnosis and therapy monitoring.

The duration of symptoms was positively correlated with the dilated apical part of the capillaries and the abnormal NVC pattern. On the other hand, no significant correlation was found between symptom duration and RPE changes, which may be surprising since RPE impartment is believed to be secondary to increased hydrostatic pressure and increased permeability, resulting in reduced RPE barrier function and fluid accumulation. Atrophic changes to the outer retina and RPE layer, developing secondary to choroidal vasculopathy are typical of chronic CSC [8]. Thus, it would appear that the longer the symptoms last, the more the RPE layer is affected. However, it is believed that the primary pathophysiology in CSC is choroidal abnormalities, therefore RPE changes are secondary and their connections disease duration may not be significant. This seems to be confirmed by the lack of significant correlations between all NVC features and the presence of RPE alteration in our study.

On the other hand, duration of symptoms was negatively correlated with SRF height, CRT and BCDVA and positively with EZ disruption. Thus, it is confirmed that the duration of disease is crucial for visual acuity and presence of subretinal fluid, and photoreceptors’ status.

Steroid is known to have been implicated in the pathological process of CSC by mineralocorticoid receptors. In spite of that there was no correlation between steroid use and BCDVA, BCNVA, AT, MI and NVC results. This may be due to the low number of steroid users in the study group (*n* = 7, 11.86%). Our findings are similar to Erol et al., who did not find a difference in capillaroscopy results between steroid users and nonusers. They suggested that the pathologic process results in choroidal vasculopathy regardless of triggering factors, such as steroids [7]. Nailfold and choroidal capillaries are similar, they are fenestrated and discontinuous. Thus, they could react similarly on the same factor.

In our study we found an interesting correlation between smoking and NVC results, different than in previous studies [14]. We found a significant positive correlation between smoking cigarettes and tortuous loops, as well as megacapillaries. Moreover, in smokers BCDVA and AT were significantly worse, therefore smoking may be regarded as the risk factor for worse visual outcome. Furthermore, smoking and the existence of CSC itself are correlated with a higher rate of chronic cardiovascular disease (CVD) occurrence in the middle-aged male population [15]. However, that connection is still under investigation [16,17,18,19,20].

The presented results seem to be promising, but further research would be required to confirm the relationship between NVC results and morphological, as well as functional findings concerning CSC patients. Our study may indicate the prospective value of NVC for the useful estimation of the episode duration and for determining in appropriate patients the subsequent treatment strategy to protect visual acuity. To our knowledge, this study is the first report assessing the relationship between NVC results and morphological, as well as functional findings concerning CSC. The limitation of our research is the relatively small size of the study group. Nevertheless, we believe that capillaroscopic imaging may be considered a useful, noninvasive new method of assessing microvascular changes in CSC and predicting the recovery of visual acuity, however, its diagnostic efficacy should be confirmed.

## 5. Conclusions

To sum up, the frequent abnormalities in NVC observed in CSC patients indicate microvascular involvement in CSC pathogenesis. The capillaroscopic pattern differs in CSC patients according to functional and morphological features, as well as general risk factors. In clinical practice, NVC could acquire a useful tool in CSC patients with diagnostic and prognostic value. However, its clinical application, and specificity of NVC abnormalities in CSC, need further studies, including a greater number of patients.

## Figures and Tables

**Table 1:** Demographics data of central serous chorioretinopathy (CSC) patients and healthy controls (HCs).

Variables	CSC (*N* = 59); *n* (%)	HCs (*N* = 53); *n* (%)
Gender (Female/Male)	Female, 14 (23.72%)	Female, 13 (24.52%)
	Male, 45 (76.27%)	Male, 40 (75.47%)
Mean Age (Years)	47.2 ± 9.4	46 ± 11.5
Place of habitation (Town/Village)	Town, 33 (55.93%)	Town, 30 (56.6%)
	Village, 26 (44.06%)	Village, 23 (43.39%)
Occupational Stress	13 (22.03%)	12 (22.64%)
Smoking	9 (15.25%)	8 (15.09%)
Steroids	7 (11.86%)	0
Hands freezing	7 (11.83%)	0
Xylomoetazolin	4 (6.78%)	0
Phosphodiesterase inhibitor	1 (1.69%)	0
Helicobacter pylori infection	2 (3.39%)	0
Hypertension	3 (5.08%)	0
Bronchial asthma	2 (3.39%)	0
Peptic ulcer disease	1 (1.69%)	0
Vitiligo	1 (1.69%)	0
Arthrosis	1 (1.69%)	0
Psoriasis	1 (1.69%)	0
Epilepsy	1 (1.69%)	0

**Table 2:** Comparison of NVC parameters in central serous chorioretinopathy (CSC) patients and healthy controls (HCs).

Variables	CSC (*N* = 59), *n* (%)	HCs (*N* = 53), *n* (%)	*p* < 0.05
NVC pattern (abnormal)	20 (33.89%)	4 (7.54%)	0.007 *
Disorganization of the capillary architecture	12 (20.34%)	4 (7.54%)	0.195
Hands freezing	7 (11.86%)	0	
Dilated apical part of capillaries	17 (28.81%)	0	
Dilated capillaries (diameter > 14 µm)	28 (47.56%)	7 (13.21%)	0.004 *
Enlarged capillaries (diameter 30–50 µm)	22 (37.29%)	0	0.007 *
Ramified capillaries and neoangiogenesis	23 (38.98%)	3 (5.66%)	0.000 *
Tortuous capillaries	38 (64.40%)	23 (43.39%)	0.651
Glomerular capillaries	19 (32.20%)	1 (1.88%)	0.000 *
Aneurysmal dilatations	6 (10.17%)	0	
Broken capillaries	3 (5.08%)	0	
Fresh capillary microhemorrhages	2 (3.39%)	0	
Megacapillaries (diameter > 50 µm)	1 (1.69%)	0	
Meandering capillaries	17 (28.81%)	15 (28.30%)	0.306
Bizarre capillaries	3 (5.08%)	0	

NVC—nailfold videocapillaroscopy. All data are expressed as *n* (%). *, statistically significant.

**Table 3:** Correlation between NVC parameters and OCT results.

Variables	RPE Alteration	PED	Hyperreflective Foci	EZ Layer Disruption	SRF Hight	CRT
NVC pattern (abnormal)	−0.0	0.1	0.1	0.2	0.2	0.2
Hands freezing	−0.1	0.1	0.2	0.4 *	0.0	−0.1
Disorganization of the capillary architecture	−0.0	0.1	0.2	0.1	0.0	0.1
Dilated apical part of capillaries	−0.0	0.0	0.1	0.3 *	0.1	0.0
Enlarged capillaries (diameter 30–50 µm)	−0.1	0.1	0.3 *	0.3	-0.2	−0.1
Diameter	−0.1	0.1	−0.0	0.0	-0.1	−0.0
Ramified capillaries and neoangiogenesis	0.1	0.1	0.3 *	0.0	0.1	0.1
Tortuous capillaries	0.0	0.0	−0.0	0.0	0.0	0.0
Glomerular capillaries	−0.8	−0.2	−0.3 *	−0.1	−0.1	−0.0
Aneurysmal dilatations	0.1	−0.1	0.0	−0.2	−0.1	−0.0
Broken capillaries	−0.1	−0.1	0.1	0.1	0.1	0.0
Fresh capillary microhemorrhages	0.1	−0.1	0.1	0.1	−0.1	−0.2
Megacapillaries (diameter > 50 µm)	0.1	−0.1	0.1	−0.1	-0.0	−0.0
Meandering capillaries	0.1	−0.1	0.3 *	0.0	0.2	0.1
Bizarre capillaries	0.1	−0.1	0.1	−0.1	0.0	−0.0

RPE—retinal pigment epithelium, PED—pigment epithelium detachment, EZ—ellipsoid zone, SRF—subretinal fluid, CRT—central retinal thickness. Spearman’s r factor, *, statistically significant.

**Table 4:** Correlation between NVC parameters and functional results.

Variables	BCDVA	BCNVA	AT	MI
NVC pattern	−0.2	0.4 *	−0.3	0.1
Hands freezing	−0.2	0.1	−0.3	0.2
Disorganization of the capillary architecture	0.1	0.2	−0.1	−0.0
Dilated apical part of capillaries	−0.4 *	0.3 *	−0.2	0.2
Enlarged capillaries (diameter 30–50 µm)	−0.2	0.2	−0.2	0.1
Diameter	−0.1	0.1	−0.2	0.1
Ramifiedcapillaries and neoangiogenesis	−0.4 *	0.1	−0.2	0.1
Tortuous capillaries	0.2	−0.0	0.5 *	−0.4 *
Glomerular capillaries	0.3 *	−0.0	0.3 *	−0.2
Aneurysmal dilatations	0.2	−0.2	0.2	−0.2
Broken capillaries	−0.1	0.2	−0.1	0.2
Fresh capillary microhemorrhages	−0.1	0.0	NA	NA
Megacapillaries (diameter > 50 µm)	0.1	−0.1	NA	NA
Meandering loops	0.0	0.2	0.0	−0.1
Bizarre loops	0.1	0.0	−0.1	0.0

Abbreviation: BCDVA—best corrected distance visual acuity, BCNVA—best corrected near visual acuity, AT—average threshold, MI—macular integrity, NA—not applicable. Spearman’s r factor, *, statistically significant.

**Table 5:** Correlation between general risk factors and NVC and functional results in central serous chorioretinopathy (CSC) patients (r Spearman’s factor).

Variables	Stress	Steroid	Smoking
Pattern of NVC	0.2	−0.1	−0.0
Hands freezing	−0.1	−0.1	−0.0
Disorganization of the capillary architecture	−0.2	0.2	0.0
Dilated apical parts of capillaries	0.3 *	0.2	−0.1
Enlarged capillaries (diameter 30–50 µm)	−0.1	−0.0	0.0
Diameter	−0.0	0.0	−0.1
Tortuous capillaries	0.0	−0.1	0.3 *
Glomerular capillaries	0.2	0.2	0.1
Aneurysmal dilatations	0.2	0.1	0.0
Broken capillaries	0.0	0.1	0.1
Fresh capillary microhemorrhages	0.1	−0.1	0.2
Megacapillaries (diameter > 50 µm)	−0.1	0.0	0.3 *
Meandering loops	−0.1	−0.1	−0.1
Bizarre loops	0.1	0.1	0.1
BCDVA	−0.3 *	−0.0	0.2
BCNVA	−0.0	0.0	0.0
AT	−0.3 *	−0.1	−0.1
MI	0.1	0.1	0.3 *

BCDVA—best corrected distance visual acuity, BCNVA—best corrected near visual acuity, AT—average threshold, MI—macular integrity. *, statistically significant.

## References

[1] Erdem Toslak I., Erol M.K., Toslak D., Cekic B., Barc Ergun M., Lim-Dunham J.E. (2017). Is the unaffected eye really unaffected? Color Doppler ultrasound findings in unilaterally active central serous chorioretinopathy. J. Med. Ultrason. (2001).

[2] Kilic D., Karahan M., Vural E., Dursun M.E., Demirtaş A.A. (2020). Abnormal retrobulbar blood flow variables in central serous chorioretinopathy. J. Clin. Ultrasound.

[3] Cutolo M., Pizzorni C., Sulli A. (2005). Capillaroscopy. Best Pract. Res. Clin. Rheumatol..

[4] Ingegnoli F., Gualtierotti R., Lubatti C., Bertolazzi C., Gutierrez M., Boracchi P., Fornili M., De Angelis R. (2013). Nailfold capillary patterns in healthy subjects: A real issue in capillaroscopy. Microvasc. Res..

[5] Kosior-Jarecka E., Bartosińska J., Łukasik U., Wróbel-Dudzińska D., Krasowska D., Chodorowska G., Żarnowski T. (2018). Results of Nailfold Capillaroscopy in Patients with Normal-Tension Glaucoma. Curr. Eye Res..

[6] Maric V., Grgurevic A., Cirkovic A., Stankovic S., Marjanovic I., Milovanovic J., Milovanovic A., Bozic M. (2019). Nailfold capillary morphology and platelet function in patients with exfoliative glaucoma. PLoS ONE.

[7] Erol M.K., Balkarli A., Toslak D., Dogan B., Durmaz D., Süren E., Altun S., Bulut M., Cobankara V. (2016). Evaluation of nailfold videocapillaroscopy in central serous chorioretinopathy. Graefes Arch. Clin. Exp. Ophthalmol..

[8] Van Rijssen T.J., van Dijk E.H.C., Yzer S., Ohno-Matsui K., Keunen J.E.E., Schlingemanne R.O., Sivaprasad S., Querques G., Downes S.M., Fauser S. (2019). Central serous chorioretinopathy: Towards an evidence-based treatment guideline. Prog. Retin. Eye Res..

[9] Smith V., Herrick A.L., Ingegnoli F., Damjanov N., De Angelis R., Denton C.P., Distler O., Espejo K., Foeldvari I., Frech T. (2020). Standardisation of nailfold capillaroscopy for the assessment of patients with Raynaud’s phenomenon and systemic sclerosis. Autoimmun. Rev..

[10] Gallucci F., Russo R., Buono R., Acampora R., Madrid E., Uomo G. (2008). Indications and results of videocapillaroscopy in clinical practice. Adv. Med. Sci..

[11] Yava G.F., Küsbeci T., Ka ikci M., Günay E., Doğan M., Unlü M., Inan U.Ü. (2014). Obstructive sleep apnea in patients with central serous chorioretinopathy. Curr. Eye Res..

[12] Borrelli E., Zuccaro B., Zucchiatti I., Parravano M., Querques L., Costanzo E., Sacconi R., Prascina F., Scarinci F., Bandello F. (2019). Optical Coherence Tomography Parameters as Predictors of Treatment Response to Eplerenone in Central Serous Chorioretinopathy. J. Clin. Med..

[13] Lee H., Lee J., Chung H., Kim H.C. (2016). Baseline spectral domain optical coherence tomographic hyperreflective foci as a predictor of visual outcome and recurrence for central serous chorioretinopathy. Retina.

[14] Yuksel E.P., Yuksel S., Soylu K., Aydin F. (2019). Microvascular abnormalities in asymptomatic chronic smokers: A videocapillaroscopic study. Microvasc. Res..

[15] Hsu H.-J., Lee C.h.-L., Chao S.C., Nien C.W., Tzeng S.H., Huang J.Y., Ko T.C., Yang S.F., Lin H.Y. (2019). The Correlation of Central Serous Chorioretinopathy and Subsequent Cardiovascular Diseases of Different Types: A Population-Based Cohort Study. Int. J. Environ. Res. Public Health.

[16] Chatziralli I., Kabanarou S.A., Parikakis E., Chatzirallis A., Xirou T., Mitropoulos P. (2017). Risk factors for central serous chorioretinopathy: Multivariate approach in a case-control study. Curr. Eye Res..

[17] Chen S.N., Chen Y.C., Lian I. (2014). Increased risk of coronary heart disease in male patients with central serous chorioretinopathy: Results of a population-based cohort study. Br. J. Ophthalmol..

[18] Tsai D.C., Huang C.C., Chen S.J., Chou P., Chung C.M., Chan W.L., Huang P.H., Chen T.J., Lin S.J., Chen J.W. (2012). Central serous chorioretinopathy and risk of ischaemic stroke: A population-based cohort study. Br. J. Ophthalmol..

[19] McAloon C.J., Osman F., Glennon P., Lim P.B., Hayat S.A., Papageorgiou N. (2016). Chapter 4—Global epidemiology and incidence of cardiovascular disease. Cardiovascular Diseases.

[20] Daruich A., Matet A., Behar-Cohen F. (2017). Central serous chorioretinopathy. Dev. Ophthalmol..

